# Research and Remedies

**Published:** 1913-09-20

**Authors:** 


### RESEARCH AND REMEDIES.

### Uterine Cancer.

A few months ago we noted in these columns the latest statistics of the results of Wertheim's operation for cancer of the uterus as it is performed by the Viennese Professor himself (The Hospital, May 31, 1913, p. 280). Put very briefly, Wertheim claims that he cures more than 18 per cent. of all the cases seen (nearly 50 per cent. of which are too far advanced for operative surgery), as judged by freedom from recurrence for at least five years; and that of those operated on and surviving the operation, 57 per cent. are "cured" in the same sense. In the *Johns Hopkins Hospital Bulletin* Professor Howard Kelly, the celebrated American gynæcologist, analyses his results in treating the same disease. He has not, it is true, had the same enormous experience as Wertheim has, but he has had quite enough to constitute him a high authority. Nor does his technique conform in every minute detail with that of Wertheim; yet it is clear that he adopted the general principles of the latter's operation at a much earlier period than most surgeons did, and has practised it consistently ever since. Professor Kelly, again, deplores the low standard of professional efficiency of a great many American doctors, as evidenced by the many advanced cases which he sees, owing to failures in diagnosis during the early and curable stage. His percentage of operated cases is nearly as high as Wertheim's; but he says that many of them were not strictly speaking operable as far as prospect of complete extirpation of the disease was concerned. Of those surviving the operation, and traced for five years subsequently, 36.7 per cent. are "cured," that is, are free from all signs of cancer; this, it will be noted, is a good deal smaller figure than that which Wertheim publishes. So also the percentage of actual accomplishment (cases "cured" out of total number seen) is 10.5 in Professor Kelly's clinic. The differences are so great that—assuming the statistics to be equally reliable in the two cases —it is probable that some other factor besides difference in the operative technique is responsible. Most probably, as Kelly suggests, failure to diagnose the disease at the earliest moment is partly the reason; and, if so, it is a formidable indictment of medical education in the United States.

### Double Second Heart Sound.

The meteoric rise of the new cardiology, which attaches far less importance to the actual valves of the heart than did the current teachings of ten and twenty years ago, has distracted a good deal of the attention that was formerly paid to the exact diagnosis of valvular lesions. Undoubtedly the condition of the muscular wall of the heart is of more significance to patient and physician than that of the valves; yet the latter also deserve careful study. In the *Medical Chronicle* Dr. Core discusses the causation of the double second sound of the heart beat which is not uncommonly audible by the stethoscope. This phenomenon may be met with in perfectly healthy persons whose hearts are quite normal. Such persons can be differentiated from those in whom the sign is pathological by attention to the following points: There is nothing in their history suggestive of cardiac disability, nor any of the etiological factors of heart disease; the heart is of normal size, and there is no accentuation of the sounds; the double sound is usually audible only at the end of inspiration, and never through the whole of the respiratory cycle; the doubling is generally rapid, and the second sound feeble. Dr. Core proceeds to express the belief that, setting aside these cases of double second sound in healthy individuals, this sign is one of the most important and certain signs of mitral stenosis which we possess. If it persists through all the phases of respiration, if it is associated even vaguely with past rheumatic disease, and, above all, if it is conjoined with accentuation of the first sound, the diagnosis of mitral stenosis is certain. The sign is early and singularly constant; it is more reliable than the presystolic murmur. Such are the conclusions of this author, who quotes some interesting cases in support of his opinions.

